# On Transducers Localization in Damage Detection by Wave Propagation Method

**DOI:** 10.3390/s19081937

**Published:** 2019-04-25

**Authors:** Adam Stawiarski, Aleksander Muc

**Affiliations:** Institute of Machine Design, Cracow University of Technology, 31-155 Kraków, Poland; olekmuc@mech.pk.edu.pl

**Keywords:** elastic wave propagation, sensors localization, SHM, damage detection

## Abstract

In this paper, the elastic wave propagation method was used in damage detection in thin structures. The effectiveness and accuracy of the system based on the wave propagation phenomenon depend on the number and localization of the sensors. The utilization of the piezoelectric (PZT) transducers makes possible to build a low-cost damage detection system that can be used in structural health monitoring (SHM) of the metallic and composite structures. The different number and localization of transducers were considered in the numerical and experimental analysis of the wave propagation phenomenon. The relation of the sensors configuration and the damage detection capability was demonstrated. The main assumptions and requirements of SHM systems of different levels were discussed with reference to the damage detection expectations. The importance of the damage detection system constituents (sensors number, localization, or damage index) in different levels of analysis was verified and discussed to emphasize that in many practical applications introducing complicated procedures and sophisticated data processing techniques does not lead to improving the damage detection efficiency. Finally, the necessity of the appropriate formulation of SHM system requirements and expectations was underlined to improve the effectiveness of the detection methods in particular levels of analysis and thus to improve the safety of the monitored structures.

## 1. Introduction

The dynamic development of material engineering and technological progress in production methods cause gaining importance of the application of new materials in engineering applications. The advanced structures like composites are characterized by high strength-to-weight and stiffness-to-weight ratios. However, the constructions made of composites are often designed with the use of the high value of the safety factors because of difficulties in accurate failure prediction. The advanced materials like composites or GLARE structures are prone to different failure forms and their number is higher than in typical metallic materials. Thus, to avoid the problems with the accurate strength prediction, in the last decades, the dynamic development of the damage detection techniques was observed. The number of non-destructive inspection techniques is growing and depends on engineering applications [1,2]. The fundamental axioms of the structural health monitoring methodology in damage detection were presented by Worden [3]. One of the main issues in the effective structural health monitoring (SHM) system is signal processing, feature extraction, and data fusion techniques [4,5]. During the past decades, extensive researches were conducted in the damage detection based on a structural dynamic characteristic of the analyzed structures [6,7,8,9]. The classification of the dynamic-based damage detection methods described as a relationship between signal frequency and possible to detection damage size was presented by Gopalakrishnan et al. [10]. However, in spite of technological progress, the accuracy and effectiveness of non-destructive methods still do no give complete satisfaction. The visual-based methods applying different inspection technologies like infrared thermography, digital image correlation, tomography, or vibration measurement by laser Doppler vibrometer (LDV) requires free access to the analyzed structure what can be difficult in some applications. The vibration-based methods based on the analysis of the natural frequencies, mode shape, damping properties, and others are by definition the global methods; thus, the sensitivity of the damage detection often leaves much to be desired. The similar difficulties one can meet in the passive methods registering natural behavior of the structure during normal service life like displacement field or acceleration. Many of them are simple to use in the autonomous damage detection systems but on the other point of view, the sophisticated techniques for threshold definition are necessary to limit the number of false alarms. One of the most promising from a usability and cost-effectiveness point of view is the elastic wave propagation method, which can be accommodated to the different structures and materials with the utilization of dedicated algorithms and computing techniques [11]. The numerical and experimental tests dealing with the wave propagation phenomenon in the isotropic and anisotropic materials confirm the serviceable of this method in the damage detection systems [12,13]. Yu et al. demonstrated the effectiveness of the wave propagation analysis in the wood utility poles what proves the versatility and possibility of matching the measurement techniques to the specific subject of analysis [14,15,16,17,18].

The several measurement techniques in the time domain were developed based on experience with different materials and defects. The pitch-catch method is usually used in the case of the relatively small area of inspection and the multipoint sensor networks [18,19]. The effectiveness of this technique was validated in the structures subjected to static and fatigue loading conditions in coupling with other methods for the damage state assessment. Detailed information about measuring apparatus used in the experimental test can be found in reference [20]. The pulse-echo method was successfully applied in marine structures and pipeline constructions [21,22]. The time reversal data analysis was proposed by Jeong et al. [23] and developed by Sohn et al. [24]. Zhao et al. [25] applied this technique in damage detection in reinforced concrete structures. One of the most straightforward damage assessment based on wave propagation phenomenon is using time-of-flight parameter [26] and a direct comparison of the signals from the intact and analyzed structures in the time domain [27]. The different statistical parameters were applied in wave propagation method to quantitatively and qualitatively assess the differences between the signal from the intact structure and signal from the currently analyzed part.

The progress in damage evaluation techniques led to the successful identification of the failure form by the wave propagation method. The numerical and experimental analysis of fatigue crack size evaluation in plates structures was presented by He et al. [28] and Stawiarski et al. [29]. Giridhara et al. [30] demonstrated the localization and quantification of the hole and corrosion with the use of a circular sensor network and triangulation method. In their brief review, one can also find other imaging techniques used for identifying damages in aluminum structures and composite laminates. The evolution of the fatigue damage in the composite specimens with hole emanating by the accumulation of the axial splitting and matrix cracking was observed by Nixon-Pearson et al. [31]. The impact damage characterization in composite laminates with the use of visualization of Lamb wave propagation by laser Doppler vibrometer was described by Toyama et al. [32]. Okabe et al. [33] characterized the delamination in CFRP composite by analysis of wave mode conversion. The finite element analysis validated by experiment with artificial delamination allows for quantitative evaluation of the delamination length. Introduction of the damage index with wave propagation path definition makes possible the accurate localization and size assessment of the delamination in flat and curved composites [34]. Understanding phenomena associated with the damage evolution in composite materials allows one to improve the strength characteristics of the structures subjected to the different loading conditions [35,36,37].

The sensors number and localization in the analyzed construction are crucial parameters in the assessment of cost and usability of the damage detection system. The several approaches were considered in the literature to obtain the optimal configuration of the SHM systems. The general approach to the optimization of sensor placement in different sensors network based on gradient descent method was presented by Akbarzadeh et al. [38]. Gao and Rose [39] applied evolutionary strategy to the optimization of sensor localization in two chosen examples. The multiple optimization strategies were proposed by Yi et al. [40] in their OSPS (optimal sensor placement strategies) toolbox focused on civil engineering applications. Ostachowicz et al. [41] prepared a review of state of the art in the optimization of sensor placement for structural health monitoring. They classified the current research in the three main groups: SHM techniques, optimization algorithms, and application demands. The authors emphasized the SHM for structures in no longer a luxury but a necessity. However, the cost of the SHM system applied in real engineering structures is still the main limitation. Many research dealing with the placement of the sensors in the vibration-based damage detection methods focuses on the solving optimization problem with the use of complicated algorithms or numerical methods for limited application. This makes difficult to validate developed methods in the real structures cases. Moreover, the relatively small effort was observed in the area of general methodology of sensors placement. The fundamental assumptions of SHM systems seem to be omitted in the considerations of damage detection. The base in the damage detection by the wave propagation method, regardless of measuring techniques (pitch-catch, pulse-echo), is comparing signals from two different state of the structures. The first signal is treated as a pattern and comes from the intact structure. The second one is the signal from the currently analyzed construction. The important issue in consideration of the vibration-based SHM systems is the requirements dealing with the accuracy, damage size and type, and others. Thus, the SHM was classified into several levels of analysis. The system of the first level confirms only the presence of the defect in the analyzed structure. The second level systems allow to localize and in some cases also allow to determine the orientation of the damage. The accuracy of the indication of the localization is different and depends on system scale, number of sensors and damage detection capability. In some cases, the indication of the defected structural element in the relatively great monitored system is satisfying. In other, detection of the small defect with dimensions equal to a few millimeters can be crucial from safety and durability point of view. It should be emphasized that most of the developed SHM methods allow for realizing the first or second level of analysis. The main task of the third level systems is to assess the size of the damage. The data from three levels of diagnosis part of the SHM system can be applied in the fourth level which is focused on the estimation of the remaining life of the structure.

Based on the literature review one can conclude that the improvement of the damage detection efficiency or sensitivity is always connected with the increase of the complexity of the proposed system. In much research, the basic assumption of the proposed damage detection method is the type or size of the considered defect. Moreover, the assumption dealing with the level of analysis is often omitted what makes difficult to use the proposed method in engineering structures. However, from a practical application point of view, the greatest challenge is to build a low-cost damage detection system that would be able to notify about a different type of structural damage without complicated procedures. The goal of this paper was to demonstrate that the importance of the particular constituents of the SHM systems like the number and localization of transducers or the damage index definition is different in particular levels of analysis. Thus, the assessment of the improvement of damage detection effectiveness should always correspond to the assumed level of analysis. The issues dealing with the utilization of the piezoelectric transducers in damage detection by elastic wave propagation method in different cases with reference to the levels classification of the SHM systems were discussed to emphasize that fulfillment of the assumed requirements is not always associated with the increase of the system scale or complexity. The expectations of damage detection systems of different levels are considered with the use of numerical and experimental examples in the pitch-catch measurement technique. The several damage index definitions were presented. The extraction of the data features was analyzed in the case of the third level system. The relation between sensors number and localization and the damage detection capability was discussed with reference to the SHM system requirements. The importance of the damage detection assumptions connected with the damage definition and the defect type and size detectability was considered and summed up.

## 2. Wave Propagation Method

**Figure 1:**
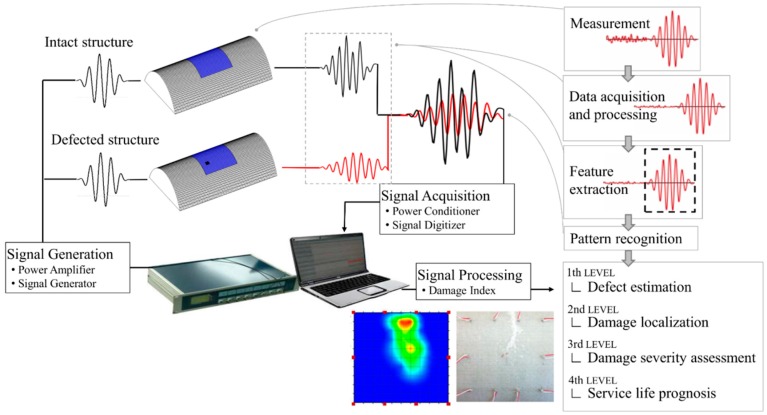
Structural health monitoring (SHM) system scheme based on wave propagation method.

The ultrasonic wave propagation method consists in transferring and analysis of the elastic wave in the thin structures. One of the most common methods of the wave generation is the use of piezoelectric transducers which can be applied as actuators and sensors in the measuring system. The small dimension of the piezoelectric (PZT) elements and relatively small cost enlarge their usability in the SHM systems. Moreover, the different shape and the form of loading cause that piezoelectric transducers can be easily fitted to the different engineering materials and structures. The schema of the damage detection system based on wave propagation method is presented in Figure 1.

On the measurement stage, one can observe the influence of environmental conditions such as temperature, noise, etc. Both the pattern signal and currently analyzed signal are prone to similar, predictable effects that can influence the final form of the measured data. Application of the specific excitation signal with defined frequency allows one to prepare the noise reduction filters which reduce the signal disturbance. The power amplifier is responsible for setting a signal amplitude, of which the level is essentially higher than the measured noise. The temperature compensation in the measurement with the use of PZT elements is also a subject of interest by researchers. Similarly to the other environmental factors, elimination of this effect from the damage detection procedure involves making preliminary tests with the use of the analyzed structure. Feature extraction of the analyzed signal consists in the registration from all measured data information valuable for damage detection procedure. The direct comparison of the pattern signal and signal from potentially damaged structure allow one to obtain the damage index value which gives quantitative or qualitative information about damage detection. Further signal processing depends on the assumed level of analysis and SHM system requirements.

### 2.1. Excitation Signal

**Figure 2:**
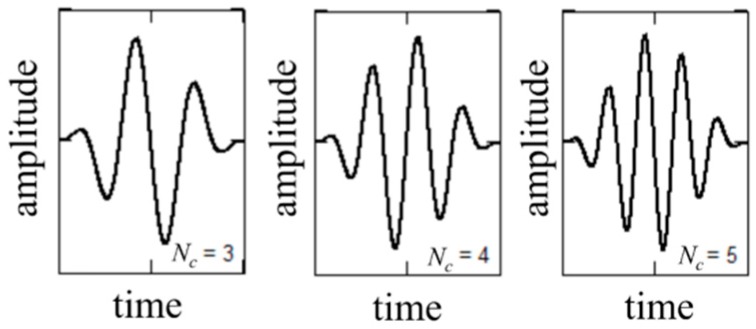
The form of excitation signal modulated by Hanning window with different numbers of cycles $N_{c}$ in wave packet.

Most studies deal with the wave propagation method using tone burst excitation signal in the form of harmonic signal multiplied by modulating window. The modulation of the excitation signal allows to concentrate the energy to the defined excitation frequency and effectively limiting the dispersion. The excitation signal modulated by a Hanning window is defined as follows:

(1) $$f\left(t\right)=\frac{1}{2}\left(1-cos\left(\frac{\omega_{0}t}{N_{c}}\right)\right)sin\left(\omega_{0}t\right),\;0\leq t\leq N_{c}/f_{0}$$

where *f*_0_ is the excitation frequency, $\omega_{0}=2\pi f_{0}$, $N_{c}$ is the number of cycles. The form of the excitation signal with a different number of cycles ($N_{c}$) in the wave packet is demonstrated in Figure 2.

The elastic wave is highly dispersive. In damage detection systems, the A_0_ (antisymmetric) and S_0_ (symmetric) modes are mostly analyzed. The utilization of the carefully chosen excitation signal allows to successfully separate particular modes and analyze only one from all possible modes. In the experimental tests of the wave propagation, the piezoelectric transducers (Noliac CMAP06) with dimensions 3 × 3 mm and 2 mm height were responsible for the signal generation and registration. The signal in the form described above was actuated by the arbitrary function generator Hameg HMF 2525 and amplified by high voltage amplifier Falco System WMA-300. The acquisition module PAQ 16000D with 16 measuring channels and 2.5 MHz of the sampling frequency was responsible for registration of the dynamic response of the structure which as processed by MATLAB environment.

### 2.2. Damage Index

The SHM system applied in the real engineering applications works autonomously. It means that the comparison of the signals from the intact and defected structures should be provided with the use of a defined procedure which would be able to extract some features of captured signals and correlate them with a different state of the structure. The damage detection and identification algorithms are based on damage index (DI) definition. The damage evolution in different materials has a stochastic character. Thus, it is difficult to predict the change of the wave form during propagation between actuator and sensor. Regardless of the measurement techniques, the arbitrary statistical measure can be applied in damage detection algorithm. The review of the statistical parameters characterizing the temporal nature of measured signals was presented by Adams [5]. However, only a few are able to include different forms of wave disturbance. One of them is the correlation coefficient defined as follows:

(2) $$\lambda_{xy}=\frac{\sum_{i=1}^{N}\left(x_{i}-\mu_{x}\right)\left(y_{i}-\mu_{y}\right)}{\sqrt{\sum_{i=1}^{N}{\left(x_{i}-\mu_{x}\right)}^{2}}\sqrt{\sum_{i=1}^{N}{\left(y_{i}-\mu_{y}\right)}^{2}}}$$

where: *N*—the number of measured discrete data, *x_n_*—the signal from the intact structure, *y_n_*—the signal from the defected structure, *μ_x_* and *μ_y_*—the mean values of the signals.

The damage index based on the correlation coefficient is often defined as:

(3) $$DI_{\lambda }\left(x,y\right)=1-\lambda_{xy}$$

**Figure 3:**
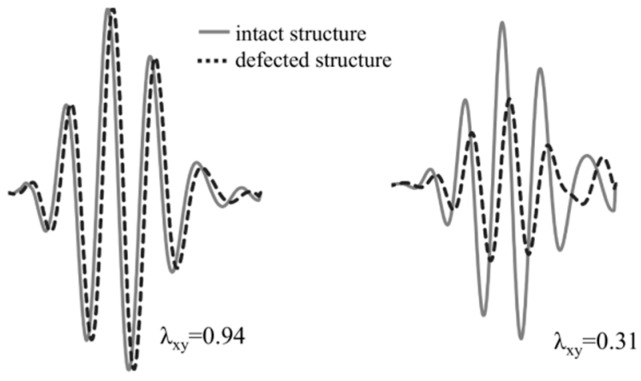
The correlation coefficient for two exemplary signals.

The correlation coefficient describes the statistical relationship between compared signals. It assumes values in the range from −1 to 1, however, in most cases of SHM applications, the initiation of the defect causes a slight signal disturbance what lead to the consideration of correlation coefficient range from 0 to 1 in practice because the signals remain in phase with the change of the amplitude or small phase shift. In the case of greater signal disturbance, especially when damage cause phase shift leading to the out of phase signals, the correlation coefficient achieves a value less than 0. In such a case, the normalization is often used to simplify the interpretation of the damage index. Figure 3 demonstrates two exemplary signals from the intact and defected structures. Two different wave disturbance can be noticed.

The small difference between signals indicates to the low probability of damage detection and the value of the damage index, in this case, is also small. In the second case, the significant signal disturbance (both shifting and amplitude change) emanates in the relatively great value of the damage index. The correlation coefficient is an effective measure in the case when signal disturbance is relatively low. When the shift of the signal of amplitude change is great, the damage index based on this statistical measure could give erroneous results. It is especially important from the durability point of view because in most cases the great wave disturbance is caused by substantial damage. To manage this situation, the area between the normalized response signal from the intact and defected structure is calculated and this value is the base for damage index definition. The area between two curves is defined as a definite integral of the difference between the discrete values of the signals:

(4) $$DI_{int}=\int_{a}^{b}\left(x_{i}-y_{i}\right)dt$$

**Figure 4:**
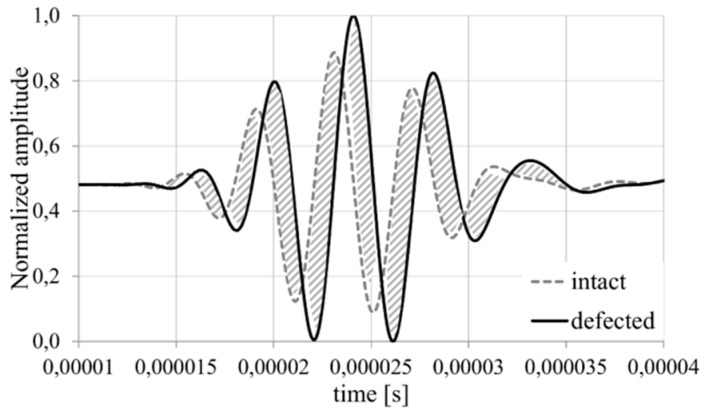
The correlation coefficient for two exemplary signals.

The area can be calculated by the numerical integration in the classical form of trapezium rule, mid-point rule, Simpson’s rule, or others. The increase of the calculated area indicates the damage evolution in the analyzed structures (Figure 4).

**Figure 5:**
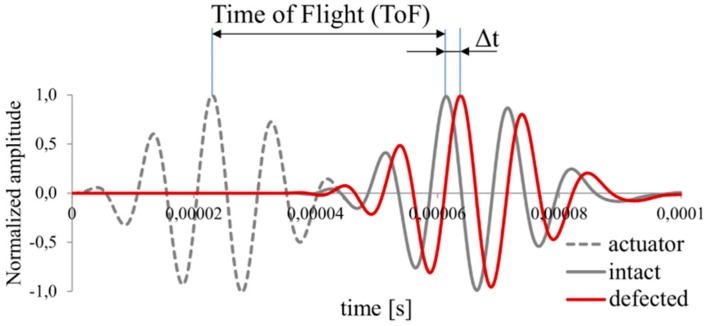
The Time-of-Flight (ToF) definition.

It is worth to point out that damage index based on the area between compared signals include not only great wave disturbance but also takes into account situation when the signal from the currently analyzed structure disappear (amplitude equal to zero). This situation may happen in the case of crack failure which precludes the wave propagation or sensor damage which also should be noticed by the SHM system. However, also this definition of damage index has the week point. In both presented damage index definitions the necessary is to assess the time window where the response signal is expected. To determine the time range where the analyzed signal should occur, the distance between actuator and sensor and the wave propagation velocity should be available for damage detection algorithm. Determination of the wave propagation in the isotropic material is quite simple and is based on the stiffness properties of the analyzed medium. However, when the elastic properties depend on the direction, such as in multilayered composite materials, determination of the wave velocity is not a trivial problem. Moreover, the great number of possible failure forms in composite structures cause that the disturbance form of the response signal may be difficult to predict. To avoid such problems the Time-of-Flight (ToF) based damage index is often used (Figure 5).

The ToF or phase shift Δt monitoring during operating of the monitored structure consists in finding the maximal value of the amplitude and analyzing of the time lag determined with the use of pattern signal. This definition of damage index is especially usable in the materials with relatively great elongation under applied loading conditions. The elongation of the wave propagation path between actuator and sensor enlarge the distance of wave propagation.

Currently, there is no universal damage index definition which would be able to include all possible cases of wave disturbances. Thus, the efficiency of the damage detection is highly dependent not only on the number and localization of sensors but also on the damage index definition which efficiency may be different for different materials and failure form.

### 2.3. Wave Propagation Path

The damage index, regardless of applied definition, has no information about the mutual position of actuator and sensors. The important conclusion is that presented forms of damage index allow building only first level system. To localize and assess the damage size, the second component of damage index definition is necessary, as it defines the wave propagation path between actuator and sensor based on geometrical parameters like coordinates in the global coordinate system. The wave propagation path in the SHM systems may be defined in different methods. The most simple is to determine the geodesic line which defines the shortest line between two points. However, this solution does not give satisfying results especially in systems that should indicate not only damage localization, but also asses the defect size. In this situation, the wave propagation path should allow generating the damage index distribution in the analyzed surface. The exemplary definition of the damage index for a second level system based on correlation coefficient with the wave propagation path defined in the form of ellipse between actuator and sensors is defined as follows:

(5) $$DI\left(x,y\right)=\overset{N}{\underset{i=1}{\sum }}\left(1-\lambda_{xy}\right)\left(\frac{\beta -R\left(\Omega \right)}{\beta -1}\right)$$

where

(6) $$R\left(\Omega \right)=\{\begin{array}{c} R_{c}\left(\Omega \right),\;R_{c}\left(\Omega \right)<\beta \\ \beta ,\;R_{c}\left(\Omega \right)\geq \beta \end{array}$$

(7) $$R_{c}\left(\Omega \right)=\frac{\sqrt{{\left(x-x_{ak}\right)}^{2}+{\left(y-y_{ak}\right)}^{2}}+\sqrt{{\left(x-x_{sk}\right)}^{2}+{\left(y-y_{sk}\right)}^{2}}}{\sqrt{{\left(x_{ak}-x_{sk}\right)}^{2}+{\left(y_{ak}-y_{sk}\right)}^{2}}}$$

$\Omega =\left[x,y,x_{ak},y_{ak},x_{sk},y_{sk}\right]$ is a function including the localization of the actuator $\left(x_{ak},y_{ak}\right)$ and sensor $\left(x_{sk},y_{sk}\right)$. As one can see, the first component of the damage index Definition (5) is responsible for quantitatively or/and qualitatively comparison of signals form the intact and defected structures. The second component defines the geometry of the SHM system. For multipoint measuring system, the damage index based on definition with information of transducers localization is calculated for each wave propagation path. The final form of the damage index distribution is determined as a sum or product of the DI obtained for all paths calculated in the classical form:

(8) $$I\left(x,y\right)=\frac{1}{N_{a}}\overset{N_{a}}{\underset{k=1}{\sum }}DI_{k}\left(x,y\right)$$

(9) $$I\left(x,y\right)=\overset{N_{a}}{\underset{k=1}{\prod }}DI_{k}\left(x,y\right)$$

## 3. Results

To discuss the transducers localization influence to the efficiency of the damage detection by wave propagation method, numerical and experimental tests were conducted. The numerical analyses of the wave propagation phenomenon in different structures were prepared in Ansys finite element analysis package. Experimental tests of static and fatigue loading condition applied to the plated structures were conducted with the use of hydraulic tension machine MTS 793A. To pay attention to the configuration of the sensors in particular structures related to the SHM system requirements the results were presented according to the level of analysis.

### 3.1. The First Level SHM Systems Results

**Figure 6:**
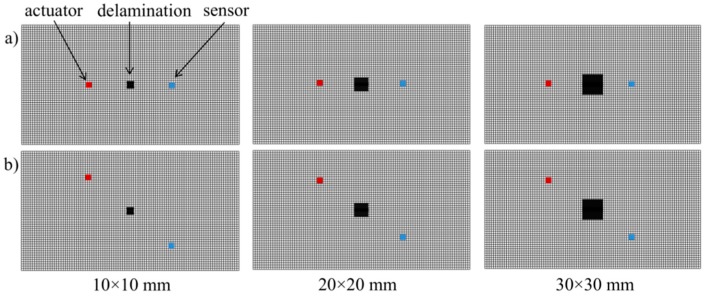
The configurations of the actuator and sensors localization where wave propagates (**a**) directly through the defect, (**b**) through the diagonal of the defect.

As was mentioned, the main goal of the first level systems is to alarm about possible damage in the monitored structures. To highlight the crucial aspects in this level of analysis the numerical analysis of the wave propagation in the thin composite structures having internal, artificial delamination between laminae layers was prepared. The epoxy/E-glass woven laminate with overall dimensions of approximately 300 × 180 mm, made of eight layers was simulated with the use of solid finite elements. The three sizes of the square delamination were analyzed. Figure 6 demonstrates the mutual position of the actuator, sensors and artificial defects for all considered cases in the pitch-catch measurement techniques. The distance between actuator/sensor and the central point of delamination was equal to 50 mm. It should be noticed that in the numerical analysis the elastic wave was generated by the normal force applied in the form described by Relation (1). In the sensor localization, the normal deflection of the structure was registered and compared with the structure without defects.

**Figure 7:**
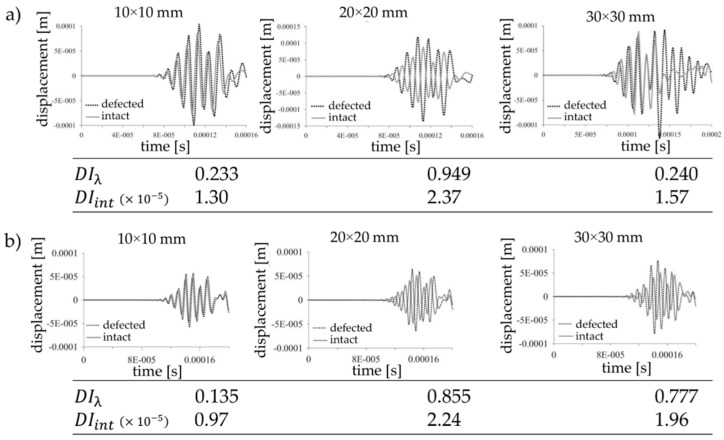
The wave propagation comparison of the intact and defected structures for the cases where wave propagates (**a**) directly through the defect, (**b**) through the diagonal of the defect and the values of damage indexes $DI_{\lambda }$ and $DI_{int}$.

Two different configuration of the actuator and sensors were analyzed. Figure 7 demonstrates the comparison of the elastic wave for the considered cases.

**Figure 8:**
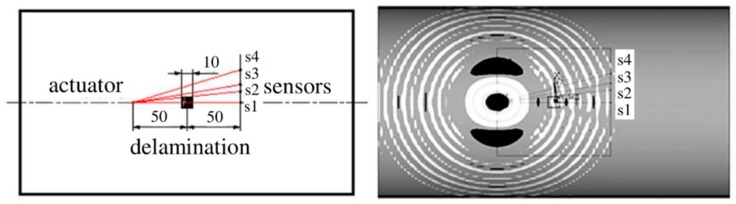
The wave propagation in the configuration with one actuator and four sensors.

The visible differences between the signal from the intact and defected structure can be observed. The wave disturbance increase with the size of the defects. It should be noticed that there are no significant differences between the analyzed configurations. Thus, even if we introduce the damage index based on arbitrary statistical measure, the efficiency of the damage detection procedure will be similar. The damage index values $DI_{\lambda }$ and $DI_{int}$ calculated after normaliation of the measured signals demonstrate the crucial problem for the first level system. The relation between the value of the damage index and the size of the defect is not as obvious as one can expect. Both damage index definitions are efficient and accurate for slight signal disturbance which in engineering applications can be observed during the initiation of the damage. When the damage size is significant with regard to the analyzed area and causes the substantial phase shift, the damage index value indicates the damage occurance but the value does not corresspond to the defect size regardless of the mutual position of the actuator and sensor. It means that in the first level system based only on a comparison of two signals the localization of the sensors in the analyzed structure is not as important, especially if we assume that the localization of the possible damage is unknown. It is worth to emphasize also that the only task of the damage index in this level is to notify about damage initiation. Let us consider a similar wave propagation case but with the use of four sensors (Figure 8).

**Figure 9:**
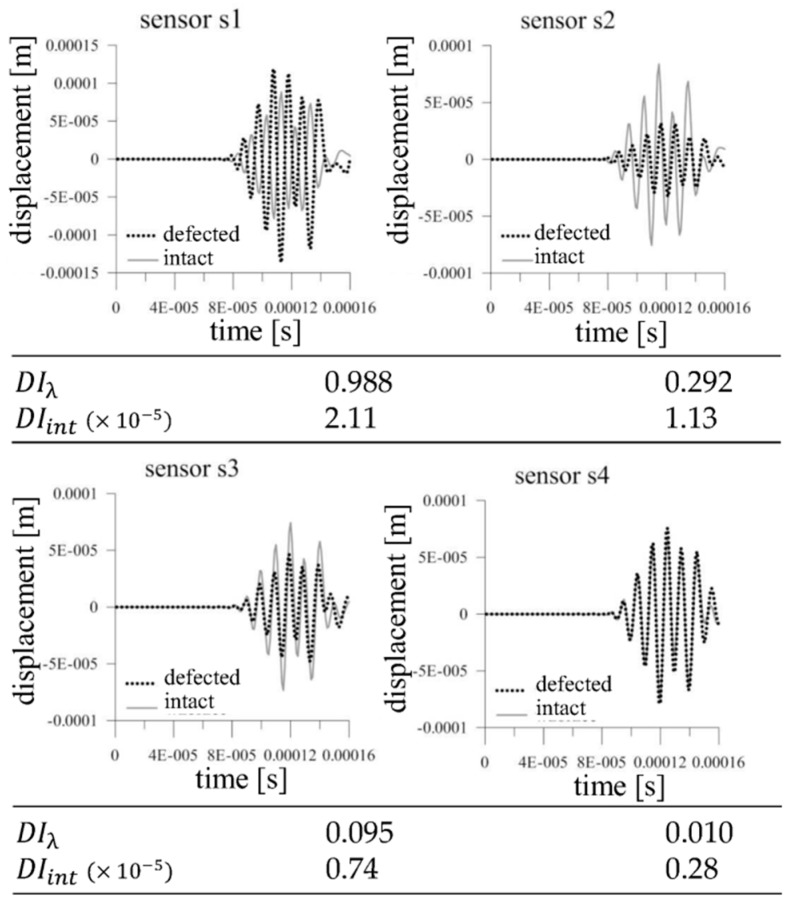
The wave propagation comparison registered in four sensors.

The internal defect cause visible wave disturbance. In the presented first level SHM system configuration the main criteria of the damage detection efficiency is the localization of the wave propagation path (red line in Figure 8). Figure 9 demonstrates the comparison of the signals from the intact and defected structures captured by four sensors which localization can be seen in Figure 8. When the wave propagation path goes directly through the damaged area the most significant difference between response signals can be observed (sensors s1 and s2). The difference between intact and defected structures measured by sensors s3 and s4 are not as important. The damage index values calculated for normalized signals correspond to the level of signals disturbance and properly indicate the damage.

It means that by introducing a greater number of sensors in the pitch-catch configuration, one can determine the damage influence zone. However, from the efficiency of the first level system point of view, there is no difference between the system with only one sensor in the localization of s1 sensor and with four sensors. Both configurations allow for obtaining information about damage occurrence. The determination of the damage influence zone or accurate localization of the defect is not the goal of the first level system. The main aspect of the design process of the first level system is not the localization of the sensors in the analyzed structures but a proper definition of the damage index which should include all possible change of elastic wave form.

### 3.2. The Second Level SHM Systems with Predictable Damage Localization

**Figure 10:**
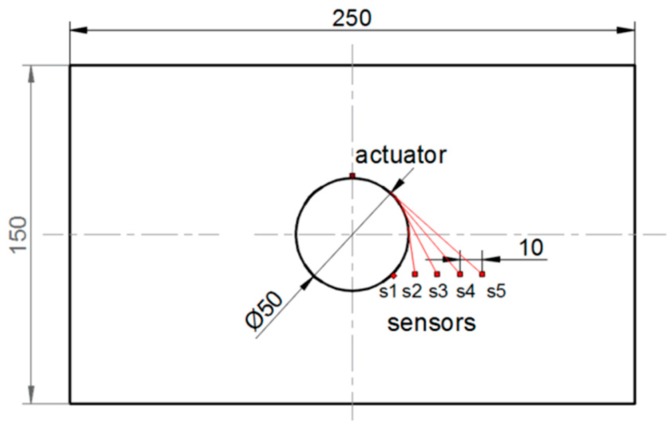
The aluminum plate with hole subjected to uniaxial tension test in the vertical direction.

The second level system allows for localization of the defect in the structures. However, in some cases, the damage localization is predictable according to the applied loading conditions and geometry of the structure. Especially in structures with different stress concentrators, the place where damage may occur is known and the main problem is to detect the damage initiation and/or monitoring of the damage growth. To discuss the localization of the sensors let us consider the uniaxial tension test of the aluminum plate with stress concentrator in the form of a circular hole. The dimensions of the analyzed plate and the configuration of the sensor placement are demonstrated in Figure 10.

**Figure 11:**
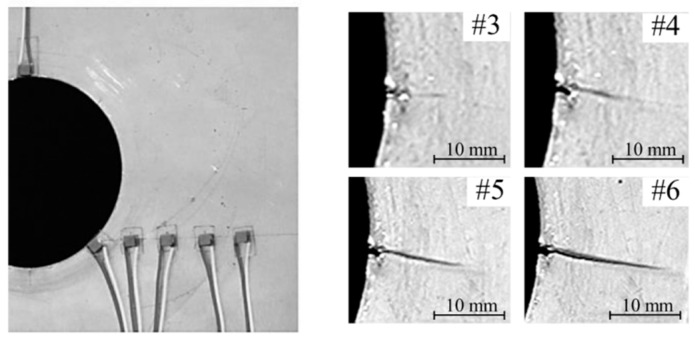
The configuration of the piezoelectric (PZT) transducers and evolution of the crack.

The thickness of the plate was equal to 2 mm. The displacement controlled loading condition with the test speed equal to 0.5 mm/min was conducted to observe the evolution of the crack at the edge of the hole. The six measurements (#1–#6) of the wave propagation were conducted with the use of piezoelectric transducers. Figure 11 presents the configuration of the PZT transducers and the evolution of the crack during the uniaxial tension test.

It should be emphasized that the actuator was placed at the edge of the hole to avoid the reflections from the hole boundaries. The crack propagated in the perpendicular direction to the loading force achieving in the final #6 stage the length approximately equal to 15 mm. The damage at the beginning of the test (#1 and #2 measurement) was almost invisible. The damage index based on correlation coefficient (see Formula (3)) and the area between two curved (see Formula (4)) was calculated. The values of the damage index based on the area between the signal measured at the beginning of the test and signal from the particular test stage (#1–#6) are presented in Table 1. The percentage value describes the area between two signals related to the structure state at the beginning of the test.

**Figure 12:**
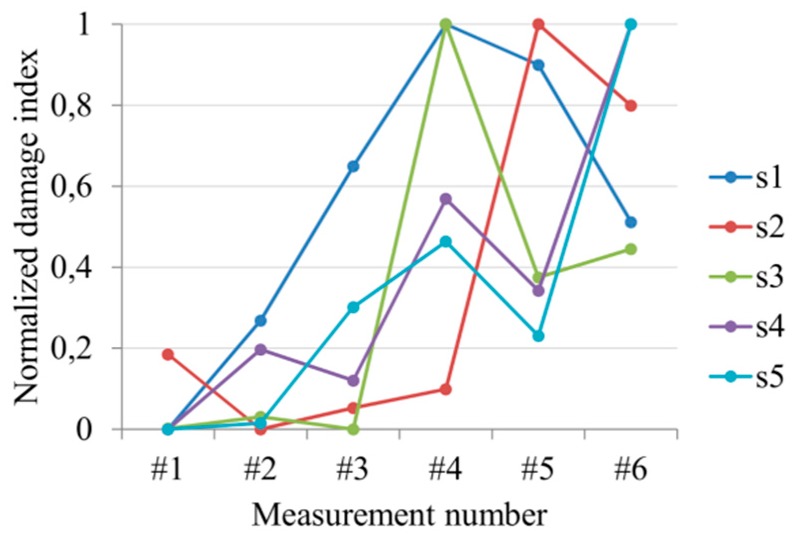
The values of damage index based on the correlation coefficient.

It can be seen that for small crack without a visible gap (#1–#3) the damage index definition can indicate erroneously results. Even when the sensor is localized at the edge of the hole (s1) and the wave propagates through the hole edge, the interpretation of the obtained results is not obvious. It is worth noting that presented sensors configuration determines the relatively small area of detection and the distance between particular transducers allow for coverage of this area by wave propagation paths. It proves that damage detection of the small cracks is difficult and is independent on the localization of the sensors. The greater crack was successfully detected by sensors in #4 measurement. Moreover, the sensors placed at some distance from the edge hole (s4 and s5) properly monitoring the evolution of the damage growth. Figure 12 presents the values of the damage index based on the correlation coefficient calculated in the same measurement points. The DI values obtained with the Formula (3) was additionally normalized according to the maximal value measured by particular sensors.

**Figure 13:**
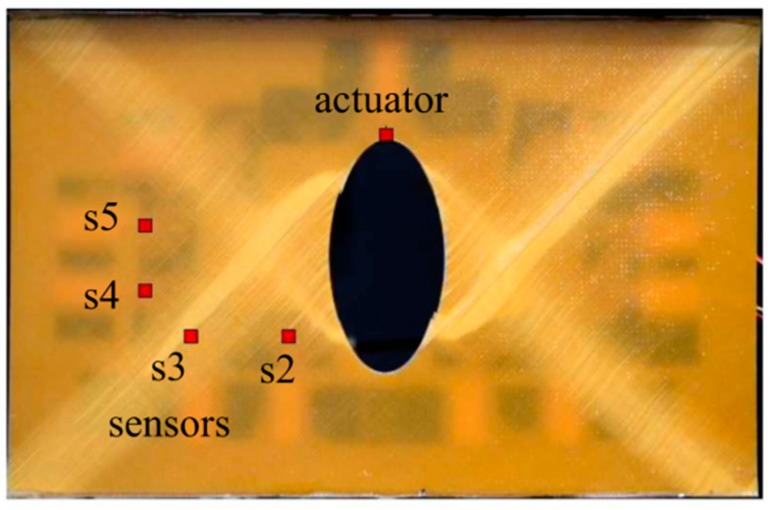
The configuration of the sensors for a composite plate with the view of failure form after N = 250,000 cycles of the fatigue test.

A general increasing tendency can be observed, which means the increasing differences between the signal from the intact and defected structures were caused by a propagating crack. Similarly to the previous damage index definition the analysis of the results from the beginning of the crack propagation is difficult. The damage index measured by sensor s1 increased from the first measurement what one can treat as a successful damage detection. However, the results from the sensors s2 an s3 indicated the damage after measurement #4 and #5. The greater structural damage is much easier to detect. As can be noticed in both presented cases, the localization of the particular sensors is less important than the lowest damage size which is possible to detect by introduced configuration. In spite of different sensors localization, in most cases, the reliable indication of the damage was obtained in the #4 measurement when the crack length was approximately equal to 10 mm. However, when the damage position is predictable, the sensors localization should be accommodated to the possible damage orientation and applied measurement technique. To discuss this conclusion, let us consider fatigue damage evolution in a composite plate with an elliptical stress concentrator. The plate was made of eight layers—epoxy/glass prepreg with unidirectional reinforcement. The symmetrical composite stacking sequence was considered—${\left[45,-45,45,-45\right]}_{S}$. The average thickness of the plate was equal to 2 mm whereas the overall dimensions was the same like in the previous case. The configuation of the actuator and sensors on the surface of the plate was demonstrated in Figure 13. The figure presents also the failure form of the plate.

**Figure 14:**
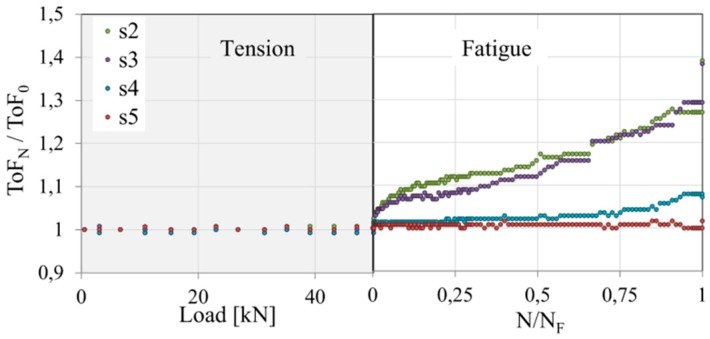
The configuration of the sensors for a composite plate with the view of failure form after 250,000 cycles of the fatigue test.

The tensile fatigue loading condition was carried out. Firstly, the plate was subjected to uniaxial tension to the mean loading force equal to 50 [kN]; then, the fatigue test was provided with the loading force amplitude equal to ±5 [kN] and the frequency equal to 30 [Hz]. The laminate withstand approximately N_F_ = 270,000 cycles before total damage. Because of symmetric angle-ply configuration application, the sensors localization was accommodated to the predictable failure form. In this case, the time of flight parameter was sequentially monitored. The results of ToF measurements during the quasi static tension to the mean load value and during the fatigue test are demonstrated in Figure 14.

The ToF measurement was related to the time of flight for the intact structures. During the quasi-static tension to the mean value of the loading force, the change of the monitored parameter was insignificant. However, just after starting the fatigue test sensors s2 and s3 registered a significant increase in the time of flight. This effect was caused by the initiation of fatigue damage in the form of matrix cracking and accumulated splits. The relatively smaller damage on the wave propagation path between an actuator and sensors s4 and s5 demonstrates the importance of the sensor localization accommodation to the failure form. Sensor s4 measured the increase of the ToF in the final stage of the fatigue damage evolution, whereas the sensor s5 was insensitive to the visible in Figure 14 matrix cracking in the wave propagation path between the actuator and that sensor.

### 3.3. The Third Level System Results

**Figure 15:**
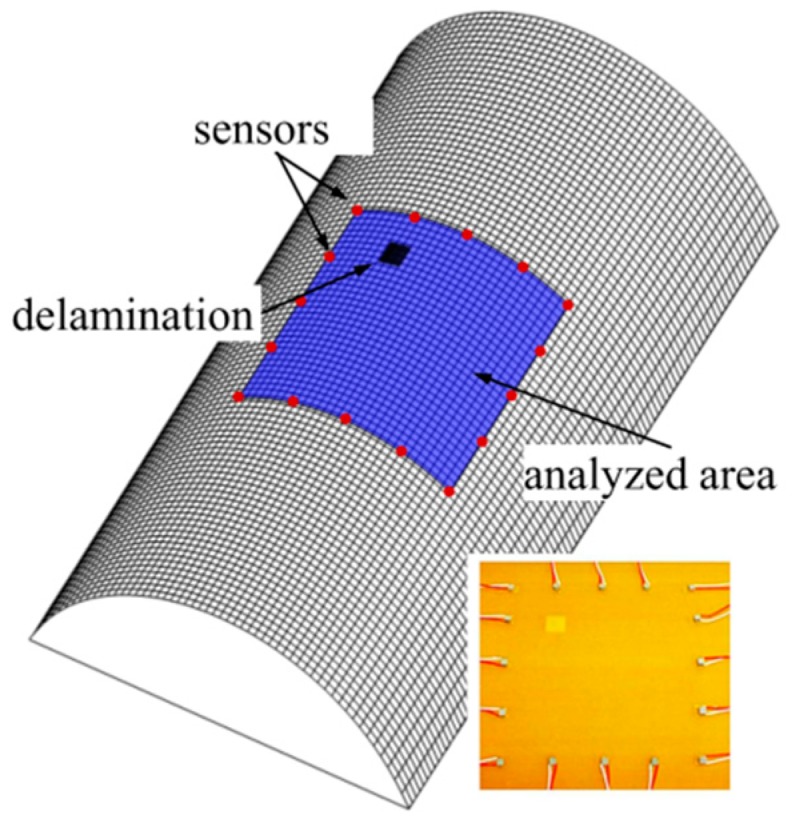
The schema of the composite cylindrical panel with delamination and the view of the PZT transducers around the analyzed area.

The third level SHM system allows not only the detection and localization of the damage in the analyzed structure, but also makes damage size assessment possible. In most cases, the multipoint measuring system is necessary to provide a detailed analysis of the damage features. The scale of the sensors network determines the size of the possible to detect a structural defect. To analyze the sensors localization in multipoint measuring system the composite cylindrical panel having single, square (10 × 10 mm), artificial delamination was considered. The total length of the panel was equal to 300 mm, the midsurface radius—92 mm and the average thickness—2 mm. The same epoxy/glass prepreg was applied in this case. However, the eight layers in the laminate were reinforced along the length of the panel. The schema of the analyzed structures and fragment of the real considered structures with applied PZT transducers is demonstrated in Figure 15.

**Figure 16:**
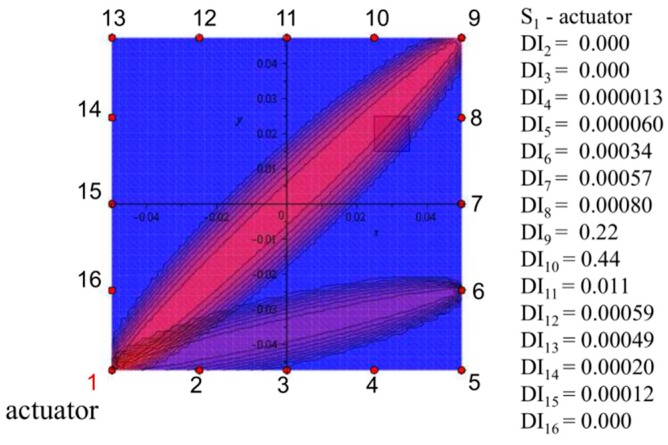
The results of the damage index calculation with marked delamination and two exemplary wave propagation paths.

The 16 PZT transducers were localized around the analyzed area. The dimensions of the analyzed area were approximately equal to 100 × 100 mm. The distance between particular sensors was equal to 25 mm. Application of the great number of the sensors allows detecting the small size defects by damage detection algorithm. The damage index based on correlation coefficient with the wave propagation path according to the Relation (4) was introduced to verify the efficiency of the multipoint measuring system. The five stages of wave propagation measurement were assumed. At one stage, one of the piezoelectric transducers generates an elastic wave and all others are responsible for registering the dynamic response of the structure. The elliptical wave propagation paths with determined damage index value are calculated and visualized in the view of the analyzed area. The results for one of the stages of wave measurement with sensors enumeration, two exemplary wave propagation paths, damage index values and schematically marked square delamination are presented in Figure 16.

**Figure 17:**
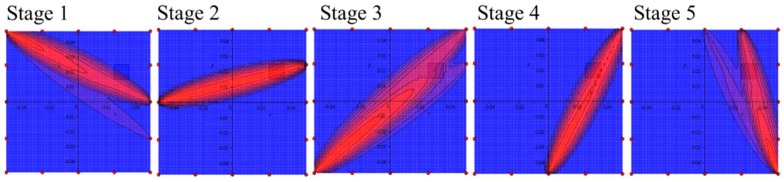
The results of the damage index distribution for particular stages of wave measurements.

The damage index based on the correlation coefficient correctly indicates the damage the comparing signals from the intact and damaged structures. The results clearly demonstrate that the application of the one actuator in multistage measurement system allows only for determining the wave propagation path where the damage index achieves the highest value. However, the precise localization of the damage is difficult in presented pitch-catch configuration. The multistage analysis is necessary to indicate the damage localization and to assess the defect size or orientation. Figure 17 presents the damage index distribution for five assumed stages with the use of summation convention defined by Relation (7).

**Figure 18:**
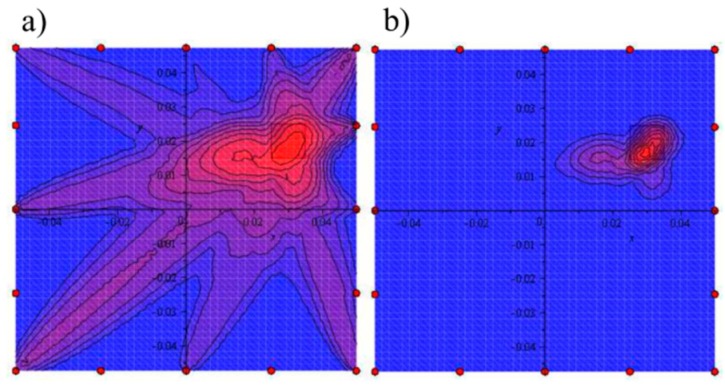
The final damage index distribution based on (**a**) Summation (7), (**b**) Conjunction (8) of the wave propagation paths.

Regardless of actuator localization, in all stages, one can observe the proper indication of the direction where the damage is expected. Thus, the efficiency of the damage detection depends mainly on the sensors number and localization. The number and distances between particular sensors in the pitch-catch measurement technique determine the damage size possible to detection. The accurate damage localization and size assessment are possible after coupling of all considered stages. Figure 18 presents the final damage index distribution calculated by summation and conjunction of all obtained wave propagation paths from particular stages. Both calculation methods indicate the delamination localization. The conjunction visualizes the common part of the wave propagation paths and is suitable for damage size assessment.

It is worth to point out that form real application point of view there are no obstacles to perform the full analysis with the number of stages equal to number of PZT transducers. However, the presented results show that even the application of a relatively low number of actuators allows building the third level system giving the satisfying results of damage detection.

## 4. Conclusions

The elastic wave propagation method is a cost-effective damage detection method that can be applied in structural health monitoring of different materials and structures. The building of the efficient damage detection system one should decide about measurement technique (pulse-echo, pitch-catch), number and localization of PZT transducers, and the parameters connected with generated elastic wave (frequency, number of cycles in the wave packet). The number and localization of the actuator and sensors are often accommodated to the analyzed structure. The geometry, the distance between transducers, and free edges or obstacles determines the analyzed area. However, the first assumption that should be established during the design process of the damage detection system is the requirements and expectations about the level of analysis. The additional important issue is what one means by structural damage. In one application even small cracks can lead to catastrophic results, whereas other, relatively large interlaminar delamination does not influence the normal service life of a structure. In the first level system, where the only expected information is the confirmation of the damage occurrence, the number and localization of the transducers are less important than the damage index definition. This parameter should be able to analyze as much as possible disturbance forms of the elastic wave. The second level system should indicate the damage localization. In the applications where the initiation of the damage is predictable because of applied loading conditions and stress concentrators, the number and localization of sensors have greater importance. In the presented cases, the transducers localization was accommodated to the expected failure form. However, even if the analyzed area determined by the mutual position of the actuator and sensors is relatively small, there is no guarantee of damage detection effectiveness in the detection of the defect initiation and defects of small dimensions. This proves that the minimal detectable defect size should be assumed during the design process of the SHM system. In the third level systems, the damage size assessment is considered. The density of the sensors network determines the possible to detect damage size. The damage detection algorithm with the proper definition of damage index, wave propagation paths and the method of the data fusion are very important in the damage detection process. In this situation, the different optimization procedures can improve the quality of the SHM system or data acquisition rate.

From all presented results, one can conclude that the main assumptions dealing with the SHM system requirements should be considered before introducing complicated procedures and optimization methods. In most engineering applications, the type and possible damage form are unknown. This makes it difficult to provide optimization procedures which very often requires objective function connected with the damage size, type, or orientation. The effectiveness of the damage detection system should be measured for a different type of damage, because from safety and reliability points of view the critical question is not how small defects can be detected but how large defects can be missed. The method of the measurement technique, number, and localization of the sensors should be accommodated to the basic assumption and SHM system requirements. It contributes to the building of cost-effective damage detection system fulfilling all assumed expectations. The usability of the optimization methods taking into account the sensors number and localization in the structures with unknown (not assumed in the objective function) damage type and size will be considered in the future works.

## Figures and Tables

**Table 1:** Percentage values of damage index based on the area between two curves calculation.

Measurement Number	Sensor Number				
	s1	s2	s3	s4	s5
#1	0.0%	28.2%	4.4%	0.0%	4.7%
#2	21.6%	0.0%	23.7%	39.8%	0.0%
#3	10.0%	37.7%	0.0%	43.7%	71.1%
#4	64.4%	99.9%	253.9%	116.5%	81.2%
#5	100.6%	227.5%	217.8%	210.8%	64.5%
#6	69.2%	187.6%	175.9%	240.8%	157.7%
